# Identification of Microbiota Biomarkers With Orthologous Gene Annotation for Type 2 Diabetes

**DOI:** 10.3389/fmicb.2021.711244

**Published:** 2021-07-09

**Authors:** Yu-Hang Zhang, Wei Guo, Tao Zeng, ShiQi Zhang, Lei Chen, Margarita Gamarra, Romany F. Mansour, José Escorcia-Gutierrez, Tao Huang, Yu-Dong Cai

**Affiliations:** ^1^School of Life Sciences, Shanghai University, Shanghai, China; ^2^Channing Division of Network Medicine, Brigham and Women’s Hospital, Harvard Medical School, Boston, MA, United States; ^3^Key Laboratory of Stem Cell Biology, Shanghai Institutes for Biological Sciences, Chinese Academy of Sciences (CAS) and Shanghai Jiao Tong University School of Medicine, Shanghai, China; ^4^Bio-Med Big Data Center, CAS Key Laboratory of Computational Biology, Shanghai Institute of Nutrition and Health, University of Chinese Academy of Sciences, Chinese Academy of Sciences, Shanghai, China; ^5^Department of Biostatistics, University of Copenhagen, Copenhagen, Denmark; ^6^College of Information Engineering, Shanghai Maritime University, Shanghai, China; ^7^Department of Computational Science and Electronic, Universidad de la Costa, CUC, Barranquilla, Colombia; ^8^Department of Mathematics, Faculty of Science, New Valley University, El-Kharga, Egypt; ^9^Electronic and Telecommunications Engineering Program, Universidad Autónoma del Caribe, Barranquilla, Colombia; ^10^CAS Key Laboratory of Tissue Microenvironment and Tumor, Shanghai Institute of Nutrition and Health, University of Chinese Academy of Sciences, Chinese Academy of Sciences, Shanghai, China

**Keywords:** type 2 diabetes, gut microbiome, machine learning, feature selection, support vector machine, microbiota biomarkers

## Abstract

Type 2 diabetes (T2D) is a systematic chronic metabolic condition with abnormal sugar metabolism dysfunction, and its complications are the most harmful to human beings and may be life-threatening after long-term durations. Considering the high incidence and severity at late stage, researchers have been focusing on the identification of specific biomarkers and potential drug targets for T2D at the genomic, epigenomic, and transcriptomic levels. Microbes participate in the pathogenesis of multiple metabolic diseases including diabetes. However, the related studies are still non-systematic and lack the functional exploration on identified microbes. To fill this gap between gut microbiome and diabetes study, we first introduced eggNOG database and KEGG ORTHOLOGY (KO) database for orthologous (protein/gene) annotation of microbiota. Two datasets with these annotations were employed, which were analyzed by multiple machine-learning models for identifying significant microbiota biomarkers of T2D. The powerful feature selection method, Max-Relevance and Min-Redundancy (mRMR), was first applied to the datasets, resulting in a feature list for each dataset. Then, the list was fed into the incremental feature selection (IFS), incorporating support vector machine (SVM) as the classification algorithm, to extract essential annotations and build efficient classifiers. This study not only revealed potential pathological factors for diabetes at the microbiome level but also provided us new candidates for drug development against diabetes.

## Introduction

Type 2 diabetes (T2D) is a systematic chronic metabolic condition with abnormal sugar metabolism dysfunction (Chatterjee et al., 2017; Zheng et al., 2018). Glucose, as an essential sugar subtype for human beings, is abnormally metabolized during T2D (Zheng et al., 2018), failing to properly transform or be stored in cells but accumulating in the circulatory system. Insulin resistance has been summarized as the general pathological cause for T2D in early studies (Goldstein, 2002; Kahn et al., 2006). However, the major concerns of T2D are not restricted to a disabled sugar storage capacity or an extremely-up-regulated blood sugar level. The complications of T2D are the most harmful to human beings and may be life-threatening after long-term durations (Schlienger, 2013). With an extremely high blood sugar, multiple diseases, including stroke and high blood pressure as blood vessel diseases (Sanahuja et al., 2016), abnormal pains and tingling as nerve dysfunctions (Yan et al., 2020), kidney damage (Bakris et al., 2020), and vision loss (Li et al., 2019), have been confirmed to be tightly correlated with long-term diabetes.

Type 2 diabetes is a common disease. According to the recent updated data, more than 27 million Americans and more than 400 million people all over the world suffered from diabetes (Bullard et al., 2018; Deputy et al., 2018). As a long-term disease, patients with pre-diabetes or early-stage diabetes may lack symptoms. One-third of all adult Americans are assumed to be in pre-diabetes status (Arthur et al., 2017). Considering the high incidence and severity at late stage, researchers have been focusing on the identification of specific biomarkers and potential drug targets for T2D. Biomarkers of diabetes at the genomic, epigenomic, and transcriptomic levels have been systematically studied (Zou et al., 2018), and multiple biomarkers, such as HbA1c (Lai et al., 2019), fructosamine (Vergès et al., 2021), and adiponectin (Liu et al., 2016), at multi-omics levels have already been identified and applied in clinical usage.

With the development of gut microbiome, the pathogenesis studies for complex diseases especially for metabolism associated diseases have been extended from traditional host genetics level to microenvironment-associated microbiome level. Microbes participate in the pathogenesis of multiple metabolic diseases including diabetes. In 2019, a summary for the microbiome role in T2D confirmed that *Bifidobacterium, Bacteroides, Faecalibacterium, Akkermansia*, and *Roseburia* can prevent the progression of diabetes, whereas *Ruminococcus, Fusobacterium*, and *Blautia* promote its development (Gurung et al., 2020). Therefore, microbial factors in gut, whose dysfunctions are underlying conditions of diabetes, may be an additional regulatory factor for the maintenance of sugar metabolism. However, current studies face two limitations in investigating the microbial influences on diabetes pathogenesis. Firstly, such studies are non-systematic, focusing on one or several significant microbes/proteins/genes. Next, they overlook the functional exploration of identified microbes, focusing only on the identification of potential diabetes-associated microbes.

To fill this gap between gut microbiome and diabetes studies, we first introduced the eggNOG (Powell et al., 2014) and KEGG ORTHOLOGY (KO) databases (Mao et al., 2005) for orthologous (protein/gene) annotations. Considering the systematic connection between KOs and related functions, these databases may help in the exploration of the potential general biological functions of identified microbes. Then, based on previously reported gut microbiome sequencing data, we applied multiple machine-learning models to identify significant microbiota biomarkers for T2D. The feature selection method, Max-Relevance and Min-Redundancy (mRMR) (Peng et al., 2005), was first applied to the data for producing a feature list. Then, the incremental feature selection (IFS) (Liu and Setiono, 1998), incorporating support vector machine (SVM) (Cortes and Vapnik, 1995) as the classification algorithm, adopted such list to extract essential annotations and construct efficient classifiers. The essential annotations can be novel biomarkers of T2D and the classifiers can be useful tools for identification of T2D samples. The identified biomarkers not only revealed potential pathological factors for diabetes at the microbiome level but also provided us new candidates for drug development against diabetes.

## Materials and Methods

### Data

We downloaded the eggNOG and KO annotations of gut microbiome in 75 T2D and 277 control from the work of Forslund et al. (2015) at http://vm-lux.embl.de/∼kultima/share/gene_catalogs/620mhT2D/620.mhT2D.RefGeneCatalog.eggnog3.annotations and http://vm-lux.embl.de/∼kultima/share/gene_catalogs/620mhT2D/620.mhT2D.RefGeneCatalog.kegg62.annotations. Within each sample, 21,902 eggNOG and 6971 KO terms were found. We aimed to investigate the functional differences of gut microbiome between T2D and normal conditions.

### Max-Relevance and Min-Redundancy (mRMR) Feature Selection

Max-Relevance and Min-Redundancy (Peng et al., 2005) is a feature selection method that can select relevant features and filter redundant features simultaneously, which has wide applications in analysis of various biological and medical systems (Zhao et al., 2018; Chen et al., 2019; Zhang S. et al., 2019; He et al., 2020; Zhang et al., 2020, 2021a). mRMR uses mutual information (MI) to estimate the feature relevance and redundancy. For variables *x* and *y*, their MI values can be computed by

(1) $$I\,\left(x,y\right)=\iint p\,\left(x,y\right)\,\log\frac{p\,\left(x,y\right)}{p\,\left(x\right)\,p\,\left(y\right)}\,d\,x\,d\,y,$$

where *p*(*x*, *y*) stands for the joint probabilistic density of *x* and *y*, whereas *p*(*x*) and *p*(*y*) stand for the marginal probabilistic densities of *x* and *y*, respectively. The mRMR method can output two feature lists, named MaxRel and mRMR feature lists, respectively. To obtain the former list, mRMR method calculates the MI value between each feature and class labels. Such list ranks features by the decreasing order of their MI values to class labels. Evidently, a feature with a high rank means that it is highly related to the class labels. However, redundancies between some top features in this list may exist. Thus, these features cannot always comprise the compact and optimum feature subspace for a certain classification algorithm. In view of this, mRMR method generates the later list, mRMR feature list, which further considers the redundancies between features. Such list is an empty one initially. Features are added to this list one by one. In each round, a feature with maximum relevance to class labels and minimum redundancies to already-selected features is selected and appended to the current list. Clearly, some top-ranked features in this list have larger feature relevance and less feature redundancy than other features. They can constitute the optimum feature subspace for a certain classification algorithm. This study only adopted the mRMR feature list because we want to build efficient classifiers for identification of T2D samples.

The mRMR program used in this study was sourced from http://penglab.janelia.org/proj/mRMR/. Default parameters were adopted to execute this program.

### Incremental Feature Selection (IFS)

Incremental feature selection (Liu and Setiono, 1998) aims to determine the optimal number of features to build a classifier for discriminating diseases, such as diabetes, by integrating a supervised classification algorithm (e.g., SVM; Cortes and Vapnik, 1995). According to the mRMR feature list generated by the mRMR method, IFS produces several feature subsets with a given interval *s* (i.e., 1 or 10). For example, the first feature subset would have the first *s* features in the mRMR feature list, then the second feature subset can have the first 2 × *s* features, and so forth. On the basis of these candidate feature subsets, a classifier can be learnt on the samples within each feature subset from the training dataset. Each classifier is evaluated by a cross-validation method (Kohavi, 1995). A classifier that can yield the best performance measurement, such as Matthews correlation coefficient (MCC) (Matthews, 1975), is found. Such classifier was called the optimal classifier in this study and the corresponding feature subset was termed as the optimal feature subset.

### SVM

Support vector machine (Cortes and Vapnik, 1995) is a supervised machine learning model for classification, which is always an important candidate for constructing efficient classifiers (Chen et al., 2017; Tahir and Idris, 2020; Zhou et al., 2020; Liu et al., 2021; Pan et al., 2021; Zhang et al., 2021b; Zhu et al., 2021). This machine can transform the original sample data with a non-linear pattern in low-dimensional space to new sample data with a linear pattern in high-dimensional space. Then, the SVM divides the data points by maximizing the point interval among different supervised classes in such a new space. Finally, SVM can predict the class label of a new sample by determining which interval this new data point belongs to.

To date, several types of SVMs have been proposed to tackle different kinds of problems. This study used the SVM optimized by the sequential minimal optimization (SMO) algorithm (Platt, 1998a,b). The tool “SMO” in Weka (Witten and Frank, 2005) implements this type of SVM and it was directly adopted in this study. It was performed with its default parameters. In detail, the kernel was a polynomial function and the regularization parameter *C* was set to one.

### Measurements

In this study, a binary classification problem (normal versus diabetes) was analyzed for each dataset with eggNOG or KO annotations. The normal samples were termed as positive samples and T2D samples were considered as negative samples. Generally, four entries: true-positive (TP), true-negative (TN), false-positive (FP), and false-negative (FN) are always counted for the predicted results of a binary classification. Accordingly, several measurements can be calculated. They are sensitivity (SN), specificity (SP), accuracy (ACC), precision, F1-measure, and MCC (Matthews, 1975; Jia et al., 2020; Liang et al., 2020; Zhang et al., 2021c), which can be computed by

(2) $$S\,N=\frac{T\,P}{T\,P+F\,N}$$

(3) $$S\,P=\frac{T\,N}{T\,N+F\,P}$$

(4) $$A\,C\,C=\frac{T\,P+T\,N}{T\,P+F\,N+T\,N+F\,P}$$

(5) $$p\,r\,e\,c\,i\,s\,i\,o\,n=\frac{T\,P}{T\,P+F\,P}$$

(6) $$F\,1-m\,e\,a\,s\,u\,r\,e=\frac{2\times S\,N\times p\,r\,e\,c\,i\,s\,i\,o\,n}{S\,N+p\,r\,e\,c\,i\,s\,i\,o\,n}$$

(7) $$M\,C\,C=\frac{T\,P\times T\,N-F\,P\times F\,N}{\sqrt{\left(T\,P+F\,P\right)\,\left(T\,P+F\,N\right)\,\left(T\,N+F\,P\right)\,\left(T\,N+F\,N\right)}}$$

The SN, SP, and precision can only evaluate the quality of predicted results in one aspect, whereas ACC, F1-measure, and MCC can fully evaluate the performance of the classifiers. Considering the fact that normal samples were much than T2D samples, MCC was selected as the key measurement in this study because it is a balanced measurement when the class sizes are of great differences. MCC has values ranging from −1 to + 1, and when MCC is equivalent to + 1, the classifier achieves the best performance.

## Results

**FIGURE 1:**
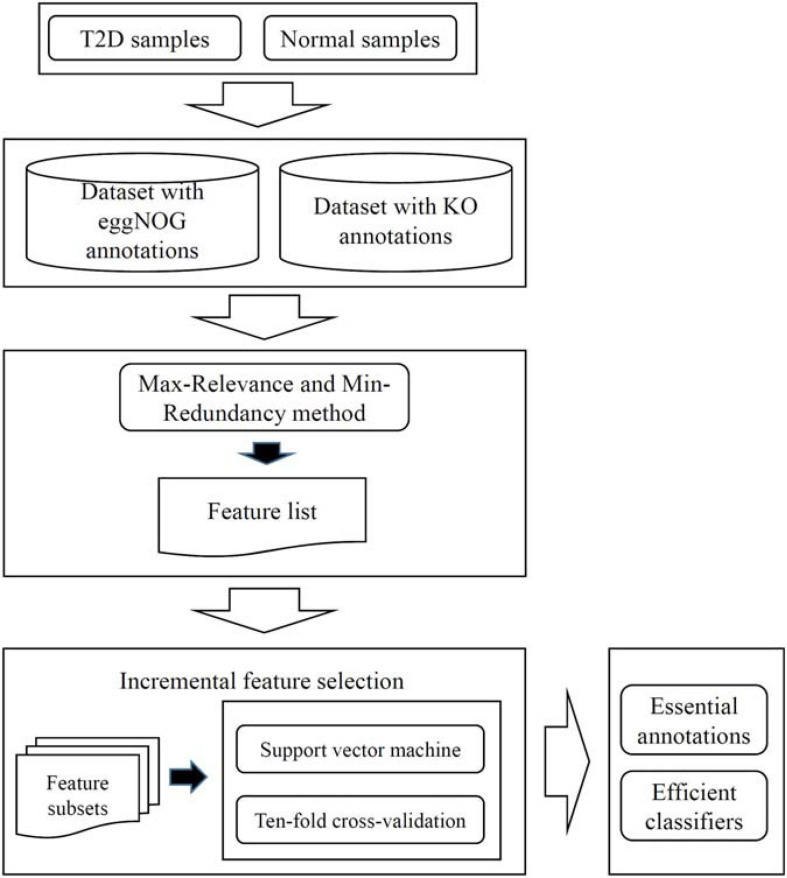
Entire procedures to investigate the T2D datasets with eggNOG or KO annotations. The dataset is first analyzed by the Max-Relevance and Min-Redundancy method, resulting in a feature list. This list is fed into the incremental feature selection method, incorporating support vector machine as the classification algorithm, to extract essential annotations and construct efficient classifiers.

Two datasets were investigated in this work: T2D microbiome data with function features from eggNOG database and T2D microbiome data with alternative function features from KO. For each dataset, a similar analysis was carried out. The whole procedures are illustrated in Figure 1. This section gives the detailed results.

### Results of mRMR Method

The mRMR method was first applied on each of two datasets to analyze the importance of each feature. A feature list, named mRMR feature list, was generated for each dataset, which is provided in Supplementary Tables 1, 2, respectively. These two lists would be used in the following IFS method.

### Results of the IFS Method

Based on an mRMR feature list, IFS was employed to give further analysis. However, there were lots of eggNOG or KO features in the corresponding dataset. If all possible feature subsets were considered, it would be time-consuming due to our limited computer power. In view of this, we designed a two-stage IFS method. In the first stage, we constructed feature subsets with an interval of 10 for each dataset. On each feature subset, an SVM classifier was built and evaluated by 10-fold cross-validation. The predicted results were counted as measurements listed in Section “Measurements.”

**FIGURE 2:**
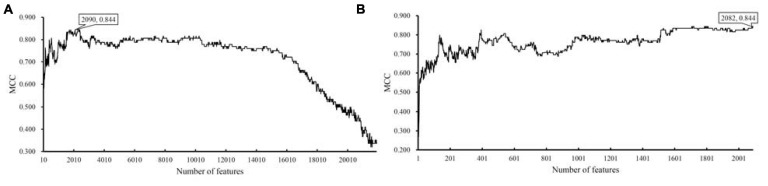
IFS curves with support vector machine (SVM) classifiers on different numbers of eggNOG features. **(A)** IFS curve with an interval of 10, the highest MCC is 0.844 when top 2090 features are adopted. **(B)** IFS curve with an interval of one, the highest MCC is still 0.844, however, it can be obtained by only using top 2082 features.

**FIGURE 3:**
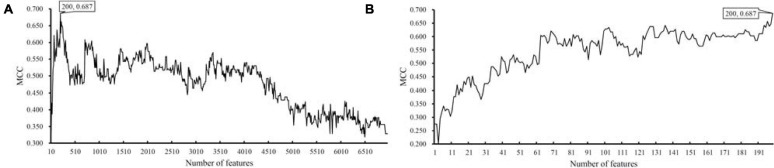
IFS curves with support vector machine (SVM) classifiers on different numbers of KO features. **(A)** IFS curve with an interval of 10, the highest MCC is 0.687 when top 200 features are adopted. **(B)** IFS curve with an interval of one, the highest MCC is still 0.687, which is obtained by the same top 200 features.

For the dataset with eggNOG annotations, obtained measurements are available in Supplementary Table 3. For an easy observation, an IFS curve was plotted, as shown in Figure 2A, where the number of features was set as *X*-axis and MCC was set as *Y*-axis. It can be observed that when top 2090 features were adopted, the SVM classifier produced the highest MCC of 0.844. As for the dataset with KO annotations, the performance of all constructed SVM classifiers is listed in Supplementary Table 4. Likewise, an IFS curve was plotted, as illustrated in Figure 3A. When top 200 features were used, the SVM yielded the highest MCC of 0.687.

**FIGURE 4:**
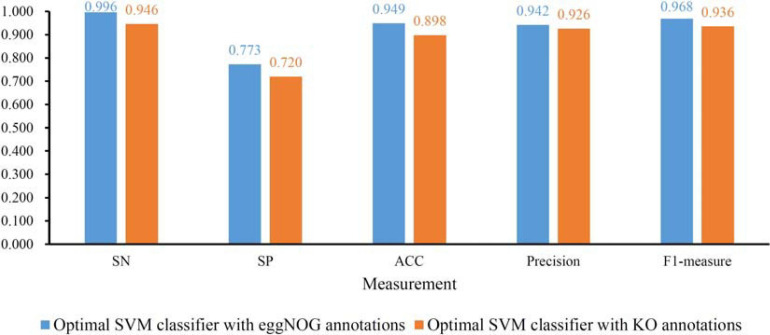
Detailed performance of the optimal support vector machine (SVM) with eggNOG or KO annotations. Except SP, other four measurements are all quite high.

To further determine the optimum feature subspace of SVM on two datasets, the second stage of IFS method was performed. For the dataset with eggNOG annotations, top 2090 features yielded the best performance of SVM in the first stage. In view of this, we did the same procedure for all possible feature subsets containing less than 2090 features. The performance of SVM classifiers on all these feature subsets is available in Supplementary Table 5. The IFS curve is displayed in Figure 2B, from which we can see that the highest MCC was still 0.844. However, it can be obtained by using top 2082 features. Thus, these features comprised the optimal feature subset and the SVM classifier with these features was called the optimal SVM classifier. The MCC of such classifier is listed in Table 1 and other measurements are provided in Figure 4. Except SP, SN, ACC, precision, and F1-measure were all quite high (>0.900). These results indicated the good performance of such optimal SVM classifier.

**TABLE 1:** MCC performance of classifiers with different features.

**Feature types**	**Number of features**	**MCC**
eggNOG	2082	0.844
KO	200	0.687

As for the dataset with KO annotations, top 200 features produced the highest MCC in the first stage of IFS method. In the second stage, all possible feature subsets with less than 200 features were considered. The performance of SVM classifiers on all these feature subsets is provided in Supplementary Table 6. Similarly, an IFS curve was plotted, as shown in Figure 3B. Interestingly, the top 200 features still yielded the highest MCC. Thus, we can determine that these top 200 features comprised the optimal feature subset. The SVM with these features was the optimal SVM classifier. The MCC yielded by such classifier is listed in Table 1 and other measurements are illustrated in Figure 4. Similar to the optimal SVM classifier with eggNOG annotations, the SP was still not very high, whereas other measurements were satisfied. It is indicated that such classifier also provided good performance.

## Discussion

As analyzed above, we constructed two optimal SVM classifiers to distinguish T2D patients from normal controls. The features used in these two classifiers can be potential biomarkers to distinguish T2D patients from normal controls at the gut microbiome level. Features describing the orthologous (proteins/genes annotation from eggNOG and KO database) with functional interpretation have been screened out and optimized to identify significant microbes together with their summarized functions associated with the pathogenesis of T2D. According to recent publications, some top functional features have been validated, and some representative features, listed in Table 2, from each database have detailed interpretations and are summarized below.

**TABLE 2:** Top annotations from eggNOG or KO databases.

**Top annotation (genes/proteins)**	**Annotation database**	**Protein/gene annotation**	**Organisms**
NOG275679	eggNOG	S-layer protein	*Bacillus thuringiensis Caldicellulosiruptor saccharolyticus Acaryochloris marina*, etc.
COG4678		Muramidase (phage lambda lysozyme)	*Escherichia coli Acaryochloris marina Burkholderia vietnamiensis*
NOG70379		ATP-binding protein	*Leptospira interrogans Helicobacter hepaticus*
NOG10530		Hypothetical protein	*Escherichia coli Burkholderia glumae*
COG0810		TonB-like protein	*Acidovorax citrulli Acinetobacter baumannii Koribacter versatilis*, etc.
K00244	KO	Fumarate reductase flavoprotein subunit	*Escherichia coli Salmonella enterica Shigella flexneri*, etc.
K14744		rzpD, prophage endopeptidase	*Escherichia coli Enterobacter cloacae Cronobacter sakazakii*, etc.
K03367		dltA, D-alanine—poly (phospho-ribitol) ligase subunit 1	*Enterobacter cloacae Pectobacterium atrosepticum Staphylococcus aureus*, etc.
K03201		virB6, lvhB6,type IV secretion system protein VirB6	*Escherichia coli Salmonella enterica Klebsiella pneumoniae*, etc.
K01006		ppdK, pyruvate, orthophosphate dikinase	*Arabidopsis thaliana Capsella rubella Eutrema salsugineum*, etc.

### Optimal Orthologous Gene/Protein Features Annotated by eggNOG Database

The first functional term identified is **NOG275679**, describing the S-layer proteins derived from multiple organisms, including *Bacillus thuringiensis* and *Caldicellulosiruptor saccharolyticus*. According to recent publications, 4-hydroxyisoleucine (4-HIL) is one of the most significant compounds for treating T2D with specific blood glucose control capacity (Zafar and Gao, 2016); 4-HIL is also a major metabolite of *B. thuringiensis* (Kodera et al., 2009; Zafar and Gao, 2016). Therefore, using NOG275679 as a biomarker may help us identify important functional microbes, such as *B. thuringiensis*, that may further assist in distinguishing diabetes patients from normal controls.

The next functional term is **COG4678** describing muramidase (phage lambda lysozyme) from multiple organisms, including *Escherichia coli*. Muramidase has been previously reported to be correlated with multiple complications of T2D. In 1989, muramidase had been shown to be correlated with tubular dysfunction (Tanaka et al., 1989), triggering diabetic nephropathy. Further in 1992, the enzyme was shown to be associated with diabetic mastopathy (Tomaszewski et al., 1992); this enzyme, which is triggered by T2D, is also associated with chronic inflammation all over the body (Maes et al., 2013). Therefore, **COG4678** is a microbe-associated biomarker for T2D.

**NOG70379** as the next identified potential biomarker describes the ATP-binding proteins in five organisms, including four *Leptospira* species and *Helicobacter hepaticus*. Given that ATP-binding proteins are essential for *Leptospira* and *H. hepaticus*, the identification of such protein from these species indicates the potential significant role of such species for T2D pathogenesis. In 2020, a systematic analysis confirmed the potential association between autoimmune disorders (including T2D) and *Leptospira* infection (Teh et al., 2020). Another independent research also validated that the infection of *Leptospira* is associated with diabetic chronic kidney disease (Carrillo-Larco et al., 2019). These tight correlations between *Leptospira* infection and diabetes support microbe **NOG70379** as a potential biomarker for T2D.

Although the next identified protein **NOG10530** is typically a hypothetical protein, the organisms from which such protein is derived from have also been linked with T2D. According to the eggNOG database, such gene/protein is mainly derived from *E. coli* and *Burkholderia glumae*. According to a systematic gut microbiome analysis on patients with T2D (Wu et al., 2017), the distributions of different strains of *E. coli* are significantly altered due to diabetes pathogenesis. Therefore, as a signature protein from *E. coli*, **NOG10530** may have a predictive potential for T2D patients.

The next identified biomarker (**COG0810**) describes the TonB-like protein in multiple organisms, including *Acidovorax citrulli*, *Acinetobacter baumannii*, and *Koribacter versatilis*. TonB-like protein is associated with the microbe-assistant vitamin B12 metabolism (Fischer et al., 1989; Gherasim et al., 2013). The majority of patients with T2D suffer from vitamin B12 deficiency (Kibirige and Mwebaze, 2013). Therefore, as a mediator for vitamin B12 metabolism, **COG0810** is a potential biomarker for distinguishing T2D patients and normal controls.

### Optimal Orthologous Gene/Protein Features Together With Functional Interpretations Annotated by KO Database

As discussed above, multiple genes/proteins from microbes have been identified and associated with T2D. For further functional exploration and summarization, we predicted another group of gene/protein features with functional annotation from KO database.

The first identified functional term **K00244** describes the fumarate reductase flavor-protein subunit. Such term has been functionally annotated with citrate cycle, oxidative phosphorylation, and carbon fixation pathways in prokaryotes. In 2019, a mouse-based study (Beli et al., 2019) confirmed that carbon metabolism, including the carbon fixation of prokaryotes, is altered during the initiation and progression of T2D. Therefore, **K00244** is predicted as a potential diabetes-associated protein at the microbial level.

The second functional term **K14744** (rzpD, prophage endopeptidase) has also been predicted to participate in distinguishing T2D patients and normal controls. Although no direct evidence confirms its pathological role for T2D, the specific role of the phage from which such protein is mainly derived from has been validated during T2D progression, participating in the regulation of chronic inflammatory environment (Górski et al., 2016; Ma et al., 2018). Therefore, K14744 may also be a potential biomarker for the identification of T2D, playing the specific role of prophage endopeptidase for phage-mediated biological processes.

The next functional term **K03367** describes the D-alanine—ploy (phospho-ribitol) ligase subunit 1, which has further been associated with the *Staphylococcus* infection and D-alanine metabolism. For staphylococci, in 2015, a research confirmed that patients with T2D have different abundances and kinds of *Staphylococcus* in the gut microbiome compared with normal controls (Gan, 2013; Farnsworth et al., 2015), indicating that *Staphylococcus* infection is associated with the initiation and progression of T2D. Further, key metabolites of D-alanine metabolism are gut microbiome markers of T2D mellitus (Wang et al., 2017), corresponding with our prediction.

Other functional terms like K03201 describing protein VirB6, and K01006 describing pyruvate, orthophosphate dikinase have also been predicted to be associated with type 2 diabetes via microbiome level regulation. Recent publications have also linked these proteins with the pathogenesis of T2D, indicating that they may also be potential biomarkers. Vir86 was identified in a long-read metagenomics exploration of human gut and is functionally correlated with inflammatory bowel disease and T2D (Suzuki et al., 2019). Pyruvate, orthophosphate dikinase is a potential pharmacological target of hypoglycemic agents (Zhang F. et al., 2019), with altered expression level from gut microbiome in response to the pharmacological effects of drugs. Therefore, these proteins with their functional annotations may be potential biomarkers distinguishing T2D at the gut microbiome level.

Overall, the identified optimal eggNOG and KO terms, which can be used to describe effective genes/proteins together with their potential biological functions and pathways, have all been associated with T2D pathogenesis and sugar metabolism-associated pathways. Therefore, on the basis of eggNOG orthologous and KO functional annotations, the machine learning models that we applied can identify optimal biomarkers or drug targets for further translational medicine studies on T2D and lay a solid foundation for studies of the detailed pathogenesis of T2D.

## Conclusion

This study investigated two T2D microbiome datasets. One was annotated by eggNOG annotations, whereas the other one was annotated by KO annotations. Several machine learning models were applied to these two datasets. Some latent biomarkers were extracted and efficient classifiers were constructed. They can be useful for identifying T2D patients from normal controls and improving our understanding on T2D at the gut microbiome level.

## Data Availability Statement

Publicly available datasets were analyzed in this study. These data can be found here: http://vm-lux.embl.de/~kultima/share/gene_catalogs/620mhT2D/.

## Author Contributions

JE-G, TH, and Y-DC designed the study. Y-HZ, WG, TZ, SZ, and LC performed the experiments. Y-HZ, TZ, MG, and RM analyzed the results. Y-HZ, WG, and TZ wrote the manuscript. All authors contributed to the research and reviewed the manuscript.

## Conflict of Interest

The authors declare that the research was conducted in the absence of any commercial or financial relationships that could be construed as a potential conflict of interest.

## Supplementary Material

Supplementary Table 1 Ranked eggNOG features by mRMR.

Supplementary Table 2 Ranked KO features by mRMR.

Supplementary Table 3 Performance of IFS for eggNOG features with an interval of 10.

Supplementary Table 4 Performance of IFS for KO features with an interval of 10.

Supplementary Table 5 Performance of IFS for NOG features with an interval of one.

Supplementary Table 6 Performance of IFS for KO features with an interval of one.
